# Insight, Narcissism and Diabetes: friends or enemies?

**DOI:** 10.1192/j.eurpsy.2023.856

**Published:** 2023-07-19

**Authors:** L. Manarte, A. Correia, A. Andrade

**Affiliations:** 1 Psychiatry and Mental Health, Faculdade de Medicina; 2 IDMEC, Instituto Superior Técnico, Lisbon, Portugal

## Abstract

**Introduction:**

Diabetes is one of the diseases in which treatment adherence is most fragile. Several factors seem to contribute to the lack of treatment compliance of the disease, from longer duration of diabetes to mental health issues. In this study, we try to identify the main factors affecting insight for diabetes (clinical, demographic, and narcissistic traits).

**Objectives:**

The main objective is to find clinical, demographic and narcisistic characteristics that differenciate good insight from poor insight diabetic patients.

**Methods:**

A cross-sectional study was developed with inclusion of 100 patients with diabetes, aged over 18 years, carried out at the Associação Protetora dos Diabéticos de Portugal (APDP). All the participants gave their informed consent. Patients were submitted to DAS (Diabetes Awareness Scale), and NPI-13 (Narcissistic Personality Inventory-13), the two most used evaluations for insight in diabetes and narcisic personality traits. The clinical and demographic factors were obtained by the records from APDP whose Ethic Comittee gave permission for this study.

**Results:**

The clinical and demographic characteristics of the sample of 100 patients with diabetes, are described in Table 4.1.

Positive and statistically significant correlations were found between HbA1c and total DAS (r = 0.420, p < 0.001), Symptom Attribution (r = 0.362, p < 0.001), Awareness of Negative Consequences (r = 0.229, p = 0.025), and Awareness of Need for Treatment (r = 0.210, p = 0.040) - Table 5. In other words, patients who were metabolically poorly controlled and had higher levels of serum HbA1c, showed higher levels of insight into the disease.

There was a statistically significant negative low-moderate affect correlation between Symptom Attribution of the DAS and the E/E (Empowerment/Explorativeness) sub score of the NPI-13 (r= -0.243, p=0.05)-Table 8.

**Image:**

**Figure:**
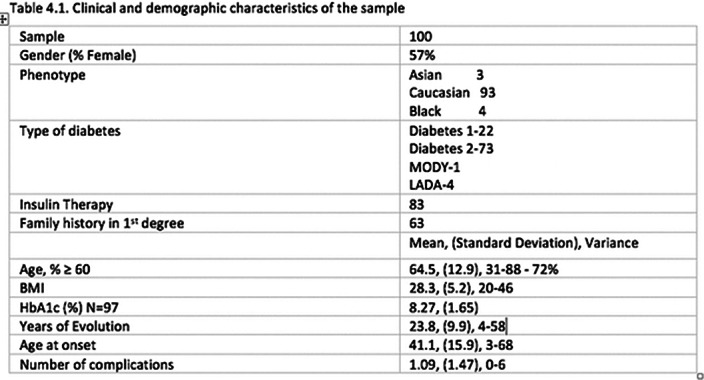

**Image 2:**

**Figure:**
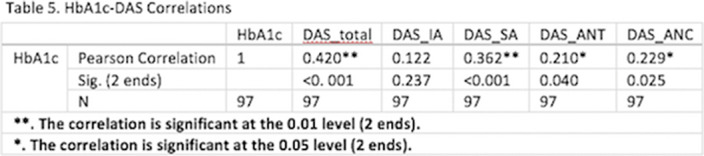

**Image 3:**

**Figure:**
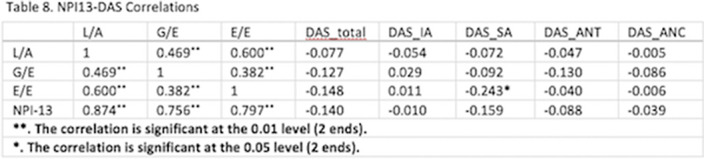

**Conclusions:**

Our results allowed us to conclude that the capacity for insight may sometimes arise in the context of already existing consequences of diabetes, in patients with poor metabolic control.Some studies had already highlighted the dubious role of increased individual perception of illness with diabetes regulation, while others were consistent with our observations, regarding the role of gender and family history in insight.

Despite these results, we propose that the knowledge of the profile of patients with insight and the anticipation of a low insight to the disease at the time of diagnosis or during follow-up allows the individualization of medical practice and the use of insight as a tool for better metabolic control of patients, ideally should arise before the development of vascular complications.

However, further studies are needed, ideally with a larger and more diverse, to understand if there are other factors that may be related to insight in the disease, as well as the development of techniques for acquisition of insight in patients with diabetes.

**Disclosure of Interest:**

None Declared

